# Does exam-targeted training help village doctors pass the certified (assistant) physician exam and improve their practical skills? A cross-sectional analysis of village doctors’ perspectives in Changzhou in Eastern China

**DOI:** 10.1186/s12909-018-1211-5

**Published:** 2018-05-11

**Authors:** Xiaohong Li, Jay J. Shen, Fang Yao, Chunxin Jiang, Fengshui Chang, Fengfeng Hao, Jun Lu

**Affiliations:** 1 Department of Health Policy and Management, China Research Center On Disability, Innovation Center for Social Risk Governance in Health, School of Public Health, Fudan University, P.O. Box 177, 130 Dong’an Road, Shanghai, 200032 China; 2 Department of Health Care Administration and Policy, University of Nevada at Las Vegas, Las Vegas, USA; 3 Changzhou Center for Disease Prevention and Control, Changzhou, China; 4 Changzhou Commission of Health and Family Planning, Changzhou, China

**Keywords:** China, Village doctors, Rural health, Training, Certified physician, Certified assistant physician, Healthcare workforce

## Abstract

**Background:**

Quality of health care needs to be improved in rural China. The Chinese government, based on the 1999 *Law on Physicians,* started implementing the *Rural Doctor Practice Regulation* in 2004 to increase the percentage of certified physicians among village doctors. Special exam-targeted training for rural doctors therefore was launched as a national initiative. This study examined these rural doctors’ perceptions of whether that training helps them pass the exam and whether it improves their skills.

**Methods:**

Three counties were selected from the 4 counties in Changzhou City in eastern China, and 844 village doctors were surveyed by a questionnaire in July 2012. Chi-square test and Fisher exact test were used to identify differences of attitudes about the exam and training between the rural doctors and certified (assistant) doctors. Longitudinal annual statistics (1980–2014) of village doctors were further analyzed.

**Results:**

Eight hundred and forty-four village doctors were asked to participate, and 837 (99.17%) responded. Only 14.93% of the respondents had received physician (assistant) certification. Only 49.45% of the village doctors thought that the areas tested by the certification exam were closely related to the healthcare needs of rural populations. The majority (86.19%) felt that the training program was “very helpful” or “helpful” for preparing for the exam. More than half the village doctors (61.46%) attended the “weekly school”. The village doctors considered the most effective method of learning was “continuous training (40.36%)” . The majority of the rural doctors (89.91%) said they would be willing to participate in the training and 96.87% stated that they could afford to pay up to 2000 *yuan* for it.

**Conclusions:**

The majority of village doctors in Changzhou City perceived that neither the certification exam nor the training for it are closely related to the actual healthcare needs of rural residents. Policies and programs should focus on providing exam-preparation training for selected rural doctors, reducing training expenditures, and utilizing web-based methods. The training focused on rural practice should be provided to all village doctors, even certified physicians. The government should also adjust the local licensing requirements to attract and recruit new village doctors.

**Electronic supplementary material:**

The online version of this article (10.1186/s12909-018-1211-5) contains supplementary material, which is available to authorized users.

## Background

Geographic discrepancies, especially urban-rural disparities, in healthcare resources and availabilities exist in China, as in other developing countries. With its economic growth during the past four decades, the Chinese government has introduced a number of measures aiming to narrow these disparities and improve healthcare in rural areas.

Based on the *Law on Physicians* issued in 1999, the *Rural Doctor Practice Regulation* [1] was issued in 2004 as a national regulation aiming to improve the quality of the healthcare workforce in rural China. The law stipulates required qualifications and related training programs and curricula for rural doctors who work in the village health clinics of rural China.

Village health clinics are always the first choice of rural residents seeking medical care [2]. They are the bottom of the rural three-tier health care network that consists of county-level health care facilities, township hospitals, and village health clinics [3]. The healthcare workforce in such clinics is composed of all the members working there [4]. The doctors, based on their qualifications, are classified as certified physicians, certified assistant physicians, rural doctors, and health workers [5].

The modern history of the training and qualifications of village doctors goes back more than 60 years, to the founding of the People’s Republic of China in 1949. That year, regulations related to qualifications for village doctors began to be created. They have evolved through four successive phases since then. The first phase was the “pre-barefoot doctor period (1949-1964)” [6]. When the Republic was established, the health system in the country was not well-developed, and the health system in rural China was especially weak. In 1951, the government put forward the *Protocol of Specific Implementation Measures for Establishing Rural Health Organizations*. It allowed some literate young people to be recruited to work at village health clinics after they received 2–6 months of medical training [6]. During this period, the focus was on establishing the village-level healthcare infrastructure, and the standard for being becoming a village doctor was low.

The second phase was the so-called “barefoot doctor boom period (1965-1980)” [6]. During this time, the concept of “*barefoot doctor*” was developed and, as a result, both the quantities and the quality of physicians were gradually improved. One was required to be trained for 3 months per year for 3 years before becoming a barefoot doctor. The number of these doctors grew to 1.46 million during this period, and they played a very important role in delivering healthcare in rural China.

During the third period (1981–1998), the “barefoot doctors” became “rural doctors.” Rapid economic growth led to a change from a “need for a doctor” to a “need for a *good* doctor.” In 1981, the government implemented a required exam specifically designed for barefoot doctors. Those who passed the exam received the certificate of *rural doctor*; those who failed only received the certificate of *village health worker*. The term “barefoot doctor” was no longer used.

**Fig. 1:**
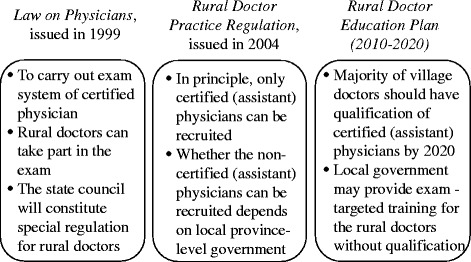
Important policies related to quality of village doctors since 1999

“Rural doctors” have become “certified (assistant) physicians” during the fourth period (1999-present). The 1999 *Law on Physicians* states that rural doctors can pass the national exams to become certified physicians or certified assistant physicians [7]. A certified assistant physician, differing from a certified physician, can provide care at the village health clinic or the township hospital independently, or provide care at a higher-level hospital under the supervision of a certified physician. In addition, a certified assistant physician who has worked for several years may take the exam to become a certified physician. Some major national laws and regulations are listed in Fig. 1.

In 2004, the *Rural Doctor Practice Regulation* [1] was passed as a supplement to the 1999 law, stipulating that “in principle, only certified (assistant) physicians can be licensed as doctors at the village health clinics” and encouraging “the existing rural doctors to take the exam and become certified (assistant) physicians.” More recently, the *National Rural Doctor Education Plan* (2010–2020) has set a goal that “the majority of village doctors should have the qualifications of certified (assistant) physicians by 2020” [8]. The Plan points out that the local government may provide physician-exam-targeted training for rural doctors who lack the qualifications of certified (assistant) physicians. Based on national statistics, however, nearly 70% of village doctors remain rural doctors in 2014 [5], indicating that it is proving to be a challenge to reach the goal of the Plan.

It is of great interest from the policy and public health perspectives whether and to what extent the *Rural Doctor Practice Regulation* and related certification exam training is helpful in encouraging and preparing rural doctors to become certified physicians. Furthermore, it is important to know whether the certification exam and the related training programs are beneficial to village doctors in regard to meeting the healthcare needs of rural residents.

Studies have shown that the quality of village doctors in rural China remains low, even after the implementation of the *Rural Doctor Practice Regulation*. Clayton and colleagues (1993) reported that inadequate sterilization during practice was common among village doctors, and that they often didn’t give adequate advice to patients who may carry infections [9]. Studies pointed out that the percentage of antibiotic prescriptions, especially intravenous injections, was very high in rural China [10, 11]. The literature suggested that over-prescribing antibiotics was very common [12], and the clinical competencies of village doctors needed improvement [13].

The literature has shown that training targeting the certified (assistant) physician exam for the graduating class of undergraduate medical students is helpful [14, 15]. However, very few studies have looked at exam-targeted training for village doctors in China, probably due to the fact that it has only been several years since the *National Rural Doctor Education Plan (2010–2020)* initiative began. Li and colleagues studied routine traditional training of village doctors in China and found that the training status and needs of rural doctors in China were disjointed [16]. Zhang and colleagues pointed out that “web-based training could be a novel pathway” [17] to remedy this deficit. Table 1 “what is already known” and “what this study adds” to this subject.

**Table 1:** “What is already known” and “what this study adds”

What is already known:	What this study adds:
✓Many studies show low quality of health services provided by village doctors.	✓This study shows low percentage of certified (assistant) physicians based on national statistics and field survey data in Changzhou.
✓Some studies are focused on shortage of and ageing in village doctors.	✓This study shows the status of participation of exam-targeted training and out-of-pocket expenditures.
✓Very few studies are on exam-targeted training.	✓This study provides suggestions on the content and methods of training of the village doctors.

## Methods

### Study design and data

This study collected data from a cross-sectional survey being conducted in July, 2012, in Changzhou City in the eastern province of Jiangsu, China. The survey was part of a study on the health workforce in village health clinics. Another part of the study dealing with the shortage of village doctors and their job satisfaction can be found elsewhere [18]. Changzhou is a prefecture-level city. While per capita GDP in 2014 was 46,629 *yuan* in the whole mainland, that of Jiangsu province was 81,874 *yuan*, ranking it fourth among the 31 provinces [19]. The per capita GDP of Changzhou in 2014 was 104,423 yuan, ranking it fourth among the 13 prefecture-level cities in Jiangsu province [20]. Three of the four rural counties/districts in Changzhou were selected for this study. All the 844 village doctors in 363 village health clinics were asked to participate, and 837 (99.17%) completed the questionnaire. In this study, “village doctors” refers to all persons working at village health clinics. They were asked about their qualifications and attitudes pertaining to the special exam-preparation programs for certified (assistant) physicians.

We also analyzed the quantity and percentage of village doctors based on longitudinal data in annual statistic books [19]. In China, village doctors consist of certified physicians, certified assistant physicians, rural doctors, registered nurses, and village health workers, depending upon their qualifications. Those who passed the exam to become rural doctors but did not take or pass both exams to become certified physicians and certified assistant physicians are termed rural doctors. Those termed village health workers are former barefoot doctors who did not pass the exam for rural doctors. Those who passed the exam for registered nurses were certified registered nurses.

The investigation was carried out in 2012 by researchers from Fudan University with help from the local municipal health bureaus, which provided the full list of the village doctors in their area. Each village doctor completed a structured questionnaire. Questions that were related to this paper consisted of: general demographic information, qualifications, reasons for failing the exam, whether the exam and exam-targeted training was helpful in their daily work, and the cost they were willing to pay for exam-targeted training (Additional file 1).

In addition to surveying village doctors, we also collected routine data about the village clinics from the annual health statistic books of 1980 to 2014. The routine data included the number of the village-level health workforce in China (called “village doctors” in this paper) and the percentage of village doctors according to their qualifications.

### Data analysis

The survey data was entered by EpiData 3.1 and analyzed using SPSS20. Data from the annual health statistic books were analyzed using Microsoft Excel 2013.

## Results

### Number of village doctors in rural China

As presented in Table 2, the number of total village doctors and number of certified (assistant) physicians increased in rural China. Though the percentage of certified (assistant) physicians gradually increased from 2005, it remained low in 2014 (20.84%). All the percentages of certified (assistant) physicians in eastern, central, and western rural China are low.

**Table 2:** Number of village doctors and percentages with different qualifications (2005–2014)^a^

Year	Total	Percentages with different qualifications (%)			
		Certified (assistant) physicians	Rural doctors	Nurse	Village health workers
2005	1,020,395	10.18	84.69	0.00	5.13
2008	1,127,531	10.61	83.22	2.20	3.97
2009	1,253,705	14.24	79.4	1.93	4.43
2010	1,292,410	13.41	79.84	2.11	4.65
2011	1,350,222	14.31	78.55	2.26	4.88
2012	1,371,592	16.97	74.58	3.23	5.22
2013	1,457,276	19.99	68.93	5.83	5.25
2014	1,460,389	20.84	67.50	6.70	4.96
Eastern	504,990	22.55	67.40	7.06	2.98
Central	546,449	22.01	66.21	7.08	4.70
Western	408,950	17.16	69.33	5.75	7.77

^a^Data are from the “National Health and Family Planning Health Statistic Yearbook (2013–2015).” The percentages in 2014 are listed according to Eastern, Central, and Western China

### Qualifications of village doctors in Changzhou

The percentage of village doctors with different qualifications is presented in Table 3. All 844 village doctors were asked about their qualifications; 837 (99.17%) responded. Only 125 (14.93%) of the 837 village doctors were certified physicians or certified assistant physicians. Only 125 (34.44%) of the 363 village health clinics had certified (assistant) physicians. The youngest village doctors (20–39 years) had the highest percentage of certified (assistant) physicians. Only 79 (9.46%) village doctors had an education level of college or higher, and 51.90% of the village doctors with an education level of college or higher were certified (assistant) physicians. However, having participated in the training was not correlated to the percentage of qualifications statistically (chi square test, *p* = 0.624).

**Table 3:** Percentage of village doctors with different qualification

Variable	N	Percentage of qualifications (%)			chi-square test^b^
		Certified (assistant) physicians	Rural doctors	Village health workers	
Total (*n* = 837)^a^	837	14.93	84.35	0.72	
Age group (*n* = 828)^a^					< 0.001
20–39 years	213	28.64	69.01	2.35	
40–49 years	191	8.90	91.10	0.00	
50–59 years	252	7.93	92.06	0.01	
60- years	172	15.11	84.30	0.59	
Education (*n* = 835)^a^					
College or higher	79	51.90	48.10	0.00	< 0.001
Medical vocational school	364	12.64	85.99	1.37	
High school	167	9.58	89.82	0.60	
Middle and primary school	225	9.78	90.22	0.00	
Working Years (*n* = 820)^a^					
0–9 years	32	34.38	62.50	3.13	< 0.001
10–19 years	256	21.48	76.95	1.56	
20–29 years	150	6.67	93.33	0.00	
30–39 years	228	11.40	88.16	0.44	
40- years	154	13.64	86.36	0.00	
Participated in training					
Yes	384	14.06	84.64	1.30	0.624
No	312	15.38	83.65	0.96	

^a^Numbers of actual responses

^b^Factors for qualifications were evaluated by chi-square test, combining the percentage of rural doctors and that of village health workers

### Attitudes of village doctors about the exam

All the 844 village doctors knew about the exam, and 748 (88.63%) had taken it. We asked those who had done so about their opinions of the exam. As shown in the Table 4, 93.72% of the village doctors stated that the exam theoretically helped improve their medical knowledge level. However, when it came to the question of “whether the exam was related to the actual health needs in rural areas,” only 49.45% of the village doctors answered “Yes.”

**Table 4:** Opinions of the exam among village doctors with different qualifications (*n* = 748)

Questions	Rural doctors (%)	Certified (assistant) physicians (%)	Total (%)
The exam impels village doctors to improve medical knowledge level theoretically*			
Yes	93.92	92.66	93.72
No	6.08	7.34	6.28
Relationship between the exam and the actual health needs in rural areas**			
Very closely	0.95	4.34	3.78
Closely	39.05	46.98	45.67
A little closely	39.05	34.72	35.43
Not closely	20.95	13.96	15.12

*Chi square test, *p* > 0.05 (two-tailed)

**Fisher exact test, *p* > 0.05 (two-tailed)

Among the 748 village doctors who had taken the exam, 625 (83.56%) did not pass; the related reasons were listed in Table 5. Leading reasons for failure were: “The written test was too difficult;” they were “too old to study;” and they were “lacking targeted training.”

**Table 5:** Reasons for failure among the village doctors who failed the exam (*n* = 613^a^)

The most important reasons for failing the exam	Numbers	Percentage (%)
Written test was too difficult	217	35.40
Too old to study	195	31.81
Technical test was too difficult	23	3.75
Lacking targeted training	65	10.60
Not meeting the prerequisite for exam	56	9.14
Too busy to study	35	5.71
Not paying much attention to the exam	5	0.82
Other	17	2.77
Total	613	100.00

^a^Number of actual responses

### Participants’ attitudes about the training

Among the 696 responding village doctors, 384 (55.17%) stated that they had attended the special exam-targeted training, whereas 312 (44.83%) had not. Those who had attended were asked about their opinions of the training (Table 6), and 3.59 and 52.60% of the respondents pointed out that the training was “very helpful” or “helpful,” respectively. As to the frequency of the training, the majority of the village doctors had attended the weekly school, however, they considered that the most effective way of training was “continuous training in school.” Further, 6.51 and 9.90% of the village doctors pointed out that the most effective way for them to study was “by internet” and “on one’s own,” respectively. Finally, 55.20% paid less than 2000 yuan for the training, while 44.80% paid more than 2000 yuan.

**Table 6:** Opinions about training among village doctors participants (*n* = 384)

Viable	Percentage (%)
Who organized the training	
Township	31.51
County	46.61
Prefecture-level city	18.23
Province	3.65
The place of training	
Township	47.40
County	34.90
Prefecture-level city	17.70
Helpfulness in preparing for the exam	
Very helpful	33.59
Helpful	52.60
A little helpful	11.46
Not helpful	2.35
Frequency of training^a^	
Having attended weekly school	61.46
Having attended continuous training for weeks in school	35.16
Having attended monthly school	14.32
The most effective way of training	
Continuous training for weeks in school	40.36
Weekly school	29.95
Monthly school	11.98
On one’s own	9.90
By internet	6.51
Other	1.30
The major content of training^a^	
Basic medical knowledge	82.29
Clinical medicine	91.93
Preventive medicine	79.95
Practical operation	79.95
Autonomy of attending the training	
Required by the township hospital	70.57
Attending the training voluntarily	29.43
Out-of-pocket expenses of the latest training (yuan)	
0–499	13.80
500–999	25.78
1000–1999	15.62
2000–2999	8.07
3000–3999	5.21
4000–4999	9.90
5000–9999	15.89
10,000–15,000	5.73

^a^More than one response possible

### Non-participants’ attitudes about the training

As shown in Table 7, according to the 312 village doctors who did not participate in the training, the leading cause of lack of participation was that they felt they were too old.

**Table 7:** Reasons village doctors did not take part in the training (*n* = 312)

Reasons	Percentage (%)
“I am too old, and I give up the exam and training.”	46.59
“The training place is too far, because there is no training in the local township.”	20.07
“I am too busy to participate in the training.”	14.77
“The costs of the training are too expensive.”	9.85
“The exam is so difficult, and I will never pass the exam. So I gave up the training.”	8.72

### Experiences and attitudes toward training according to qualifications

As shown in Table 8, all the village doctors, including both certified (assistant) physicians and rural doctors, felt the training was essential for passing the exam. They believed that the government should be mainly responsible for the expenses of the training. The majority of the rural doctors (89.91%) were willing to participate in the training in the future and 96.87% stated that they could afford up to 2000 yuan for the training.

**Table 8:** Attitudes about training among village doctors of different qualifications (%)

Item	Certified (assistant) physicians (*n* = 105) (%)	Rural doctors (*n* = 575) (%)
Have participated in the training*		
Yes	52.94	55.35
No	47.06	44.65
The training is essential for the exam.*	95.24	86.96
The government should be mainly responsible for the expenses of the training.*	73.33	66.09
Willing to participate in the training in the future	–	89.91
Maximum out-of-pocket payment for the training (yuan)	–	–
0–499	–	45.21
500–999	–	26.09
1000–1999	–	25.57
2000–5000	–	3.13

*Chi square test, two-tailed, *P* > 0.05

## Discussion

### Major problems in the exam and exam-oriented training

Our findings indicated that the percentage of certified (assistant) physicians among village doctors in Changzhou was below 20%, far from reaching the goal of “the majority of village doctors should have the qualifications of certified (assistant) physicians by 2020” that has been set by the government. More than 80% of the doctors did not pass the exam, and only about half of them felt that the exam was closely related to the actual health needs of rural residents. A little over half of the village doctors attended the exam preparation program, more than 85% of whom think the program was helpful. The majority of the non-certified (assistant) physicians (90%) were willing to participate in the exam-preparation program and were willing to pay fees up to 2000 yuan.

However, we found that the training is of limited use in increasing the percentage of certified (assistant) physicians in village clinics who pass the certification exam. There were two possible explanations. First, the percentage of those participating in the training was low, due to the fact that many village doctors thought they were too old to become certified. Barriers in attending the training program, such as inconvenient locations and high fees, only worsened the problem of low enrollment. Second, some village doctors left their village clinics after they became a certified physician, because working at village clinics was not as appealing as working in healthcare settings at higher levels [18]. It is challenging to keep certified assistant physicians at village clinics [21].

### Needs for local government actions

Our results showed that only about 1/5 of village doctors were certified (assistant) physicians in eastern China. The percentage of certified (assistant) physicians in Changzhou was even lower than that in eastern China, which is consistent with the literature stating that few village doctors leave village clinics in more economically developed areas [18]. It is important to note that the national policy of public health equalization, which began implementation in 2009, has significantly increased the tasks and workload of village doctors, which, in turn, aggravates the problem of the shortage of village doctors [18, 22, 23].

China is facing a dilemma between *quantity* and *quality,* resulting from the public health equalization policy. On the one hand, more village doctors are needed to implement the policy of basic public health equalization; on the other hand, the *Law on Physicians* and the *Rural Doctor Practice Regulation* set strict requirements for the qualifications for becoming village doctors, which seem to be barriers to converting or recruiting more village doctors.

To resolve this dilemma, it may be necessary to rethink the tasks of village health clinics. In general, they can be divided into two categories: clinical services being provided by the certified (assistant) physicians or other clinical staff, and administrative tasks that can be done by non-clinical staff. Many of the administrative tasks include data entry into computers and contacting patients for follow-up on chronic disease management. Nevertheless, village doctors currently are responsible for all these types of administrative work, which occupies a lot of their time that otherwise could be used to provide clinical services. It appears that a dual personnel model could work more efficiently than does the current system. That is, the certified (assistant) physicians or rural doctors are responsible for the clinical services, whereas non-clinical staff may be hired to take care of administrative tasks. The government may issue minimum qualifications for administrative staff to work at village health clinics.

**Fig. 2:**
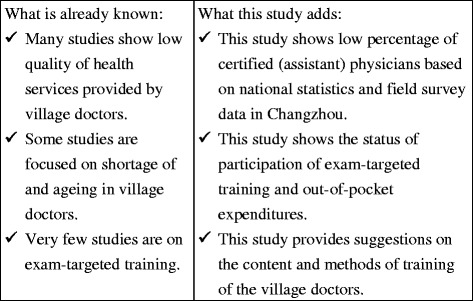
Health workforce in village health clinics

The *Rural Doctor Practice Regulation* (2004) stipulates that “in order to meet the need for human resources...the local province-level government can formulate specific regulations of licensing based on the local conditions” [1]. In other words, the local government can determine the qualifications of workers at village clinics. It seems clear that in order to alleviate the problem of doctor shortages in rural areas, local governments may need to revise their current rules and policies for staffing village clinics. More specifically, it may want to allow village health clinics to recruit non-clinical personnel. Currently, in many provincial governments, especially in more economically developed areas, such as Zhejiang [24] and Jiangsu [25], being a certified (assistant) physician is a prerequisite to obtain a license at village health clinics. In contrast, less economically developed provinces, such as Shanxi [26] and Jiangxi [27], allow persons with certain clinical training but who are not certified (assistant) physicians, yet, to practice as village doctors at village clinics. It should be feasible for more-developed provinces to learn from less-developed provinces in this regard in order to solve the doctor-shortage problems in their own rural areas (Fig. 2). Meantime, they should also provide guidelines and qualifications for hiring health workers for village clinics.

### Meeting the different training needs of village doctors

In terms of the goals of training, the local government should develop two training programs. One may be called the *physician exam preparation program* (*ProgramI*), focusing on helping non-certified (assistant) physicians pass the exam, while the other may be called the *rural practice focus program (ProgramII*), focusing on improving the practical skills of village doctors. As shown in Table 9, only rural doctors who are *potential physicians* should participate in *ProgramI*. Those rural doctors who do not want to obtain licensure to become certified (assistant) physicians do not need to participate in the exam preparation program (*ProgramI*).

**Table 9:** Different kinds of village doctors and needed training programs

Village doctors	Exam preparation program (*ProgramI*)	Rural practice focus program (*Program II*)
Certified (assistant) physician	×	√
Rural doctors —*potential physician*	√	√
Rural doctors —*non-potential physician*	×	√
Health workers	×	√

*ProgramI*will be more likely to be implemented effectively if the following issues are addressed. First, based on the fact that only 3.13% stated that they could afford up to 2000 yuan for the training, the government should reduce out-of-pocket fees for the training to encourage more participants among village health clinic workers. Second, new educational models, including an online delivery model, needs to be developed and integrated into the educational program. This will accommodate village doctors who are too busy to participate in the face-to-face training. Third, the government may use some indicators, such as education level, age, professional skill, and willingness to learn, to identify *potential physicians.*

*ProgramII* aims to improve all types of village doctors’ skills to deal with emergent medical conditions, which is included in village doctor training in other countries. Tolhurst and colleagues report that rural doctors in Australia need more training related to emergency medicine [28]. Furthermore, such training should be responsive to the fact that some emergencies, such as pesticide poisoning and dog bites, are rare in urban areas but more common in rural areas in China [29, 30].

## Limitations

This study had some limitations. First, it relies on self-reported data, and recall bias might exist, in particular when information about the licensure exam and participation in the training program is being collected. Second, the field survey was only conducted in Changzhou city in eastern China, which limits the generalizability of our findings. Third, we only collected the subjective attitudes of the village doctors; more objective information such as the village doctors’ licensure exam score was not collected.

## Conclusions

Although the licensure exam preparation training for certified (assistant) physicians is provided to increase the exam’s passing rate, the current model has had only limited success. Therefore, a dual training program model to fit the needs of different types of village doctors is more likely to be effective. The exam-preparation program fits for rural doctors who can potentially become certified (assistant) physicians, whereas the rural practice targeted program may fit well for all types of the village doctors. Measures for optimizing the first program include more carefully identifying potential certified (assistant) physicians among rural doctors, reducing the out-of-pocket training fees and developing online training modules.

## Additional file

Additional file 1: Questionnaire of village doctors in Changzhou. (DOCX 24 kb)
