# Supplementary material for: Does exam-targeted training help village doctors pass the certified (assistant) physician exam and improve their practical skills? A cross-sectional analysis of village doctors’ perspectives in Changzhou in Eastern China
Source: BMC Med Educ. 2018 May 11;18:107. doi: 10.1186/s12909-018-1211-5 (PMC5948688; doi:10.1186/s12909-018-1211-5)
Supplement: Supplementary file 1 — Questionnaire of village doctors in Changzhou. (DOCX 24 kb) [file 12909_2018_1211_MOESM1_ESM.docx]

**Questionnaire of Village Doctors**

Name: NO.

Address：

Date: Investigator

| Questions (Please fill the blanks in Right column) | Answer |
| --- | --- |
| 1. Village clinic: county township village |  |
| 1. Age (years): |  |
| 1. Education : (1) college or above (2) vocational high school (3)high school (4)middle school (5)elementary school (6) no school or not finish elementary school |  |
| 1. Qualification: (1) rural doctors (2) certified physicians (3) assistant certified physicians (4)village health workers (5)none |  |
| 1. Working as a doctor for years |  |
| 1. The most important reasons for failing the certification exam 2. Written test was too difficult 3. Technical test was too difficult 4. Lacking targeted training 5. Too old to study 6. Not meeting the prerequisite for exam 7. Too busy to study 8. Not paying much attention to the exam 9. Other |  |
| 1. Have you ever taken part in the exam-targeted training (1)yes (2)no |  |
| 1. If you haven’t taken part in the exam-targeted training, the reasons are 2. “The exam is so difficult, and I will never pass the exam. So I gave up the training.” 3. “The costs of the training are too expensive.” 4. “I am too busy to participate in the training.” 5. “I am too busy to participate in the exam.” 6. “The training is useless for the exam.” 7. “I am too old, and I give up the exam and training.” 8. “The training place is too far, because there is no training in the local township.” 9. Other reason |  |
| 1. The level of the organization who organized the latest training 2. Township 3. County 4. Prefecture-level city 5. Other |  |
| 1. The place of the latest training 2. Township 3. County 4. Prefecture-level city 5. Other |  |
| 1. The Frequency of the latest training 2. Having attended continuous training for weeks in school 3. Having attended weekly school 4. Having attended monthly school 5. Other |  |
| 1. The major content of training* 2. Basic medical knowledge 3. Clinical medicine 4. Preventive medicine 5. Practical operation 6. Other |  |
| 1. Autonomy of attending the training 2. Required by the township hospital 3. Attending the training voluntarily |  |
| 1. Out-of-pocket expenses of the latest training (yuan) |  |
| 1. Helpfulness in preparing for the exam 2. Very helpful 3. Helpful 4. A little helpful 5. Not helpful |  |
| 1. The most effective way of training 2. On one’s own 3. By internet 4. Continuous training for weeks in school 5. Weekly school 6. Monthly school 7. Other |  |
| 1. Is the training essential for the exam? (1)yes (2)no |  |
| 1. Are you willing to participate in the training in the future? (applicable to the non- certified (assistant) physician) (1)yes (2)no |  |
| 1. Relationship between the exam and the actual health needs in rural areas 2. Not closely 3. A little closely 4. Closely 5. Very closely |  |
| 1. Does the exam impel village doctors to improve medical knowledge level theoretically? (1)yes (2)no |  |
| 1. Do you believe that the government should be mainly responsible for the expenses of the training? (1)yes (2)no |  |
| 1. In your opinion, the maximum out-of-pocket payment for the training is (yuan) |  |
